# Supplementary material for: Glucose metabolism induced by Bmp signaling is essential for murine skeletal development
Source: Nat Commun. 2018 Nov 16;9:4831. doi: 10.1038/s41467-018-07316-5 (PMC6240091; doi:10.1038/s41467-018-07316-5)
Supplement: Supplementary file 1 — Supplementary Information [file 41467_2018_7316_MOESM1_ESM.pdf]

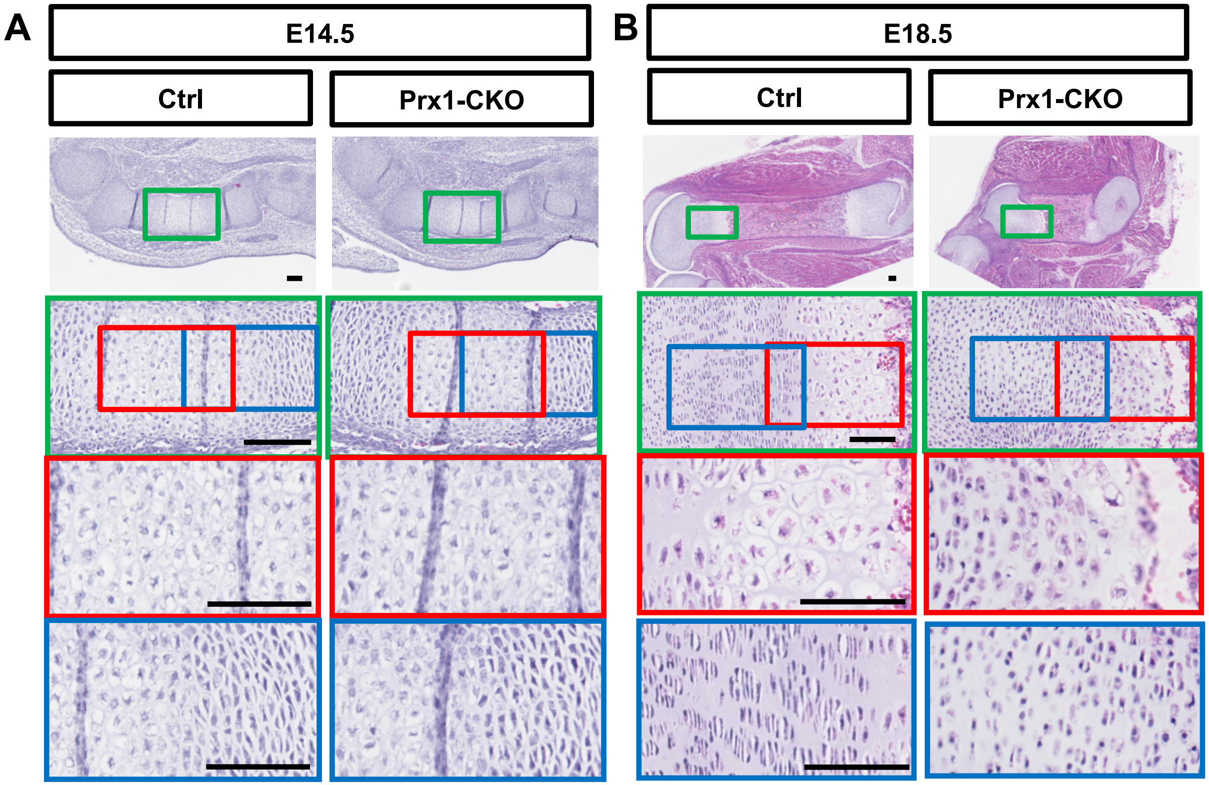

Supplementary Fig. 1. Glut1 is essential for chondrocyte maturation and hypertrophy. H&E staining of the femur section from control (Ctrl) versus mutant (Prx1-CKO) littermates at E14.5 (A) or E18.5 (B). Color-coded boxed areas are shown at higher magnification. Scale bar: 100 µm.

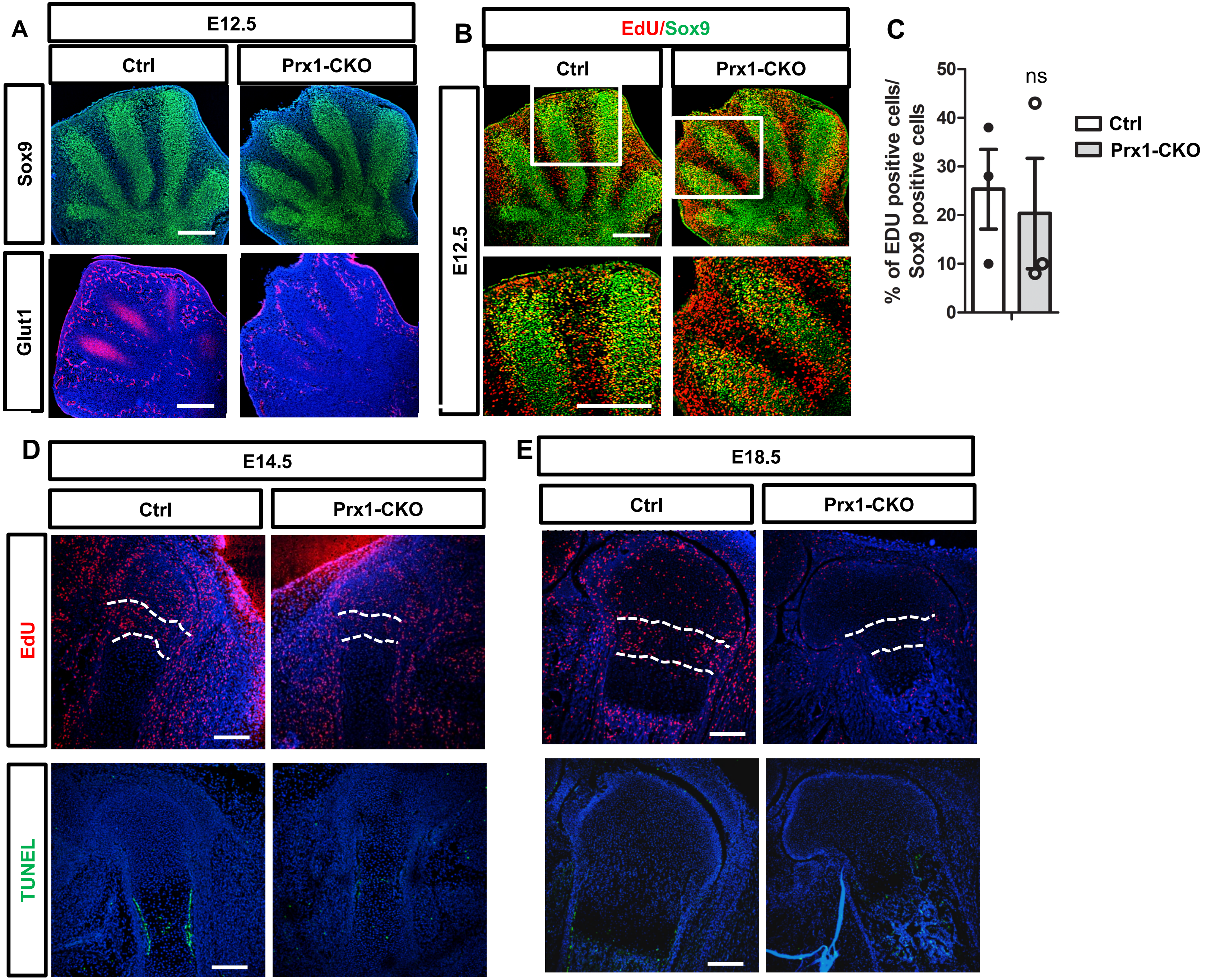

A
E12.5
Ctrl
Prx1-CKO
Sox9
Glut1
B
EdU/Sox9
Ctrl
Prx1-CKO
E12.5
C
% of EDU positive cells/
Sox9 positive cells
50
40
30
20
10
0
ns
Ctrl
Prx1-CKO
D
E14.5
Ctrl
Prx1-CKO
EdU
TUNEL
E
E18.5
Ctrl
Prx1-CKO

Supplementary Fig. 2. Glut1 is necessary for chondrocyte proliferation but not survival. (A) Immunofluorescence staining of Sox9 (up) and Glut1 (bottom) on sections of the autopod from E12.5 ctrl (left) and Prx1-CKO (right) littermates. (B) Double fluorescent labeling of EdU (red) and Sox9 (green) on sections of the autopod from E12.5 ctrl (left) and Prx1-CKO (right) littermates. Boxed areas are shown at higher magnification below and used for quantification. (C) Quantification of EdU percentage among Sox9+ cells. n=3 mice per genotype. Two-tailed Student's t test, ns : $p>0.05$. (D, E) Representative images of EdU (red) or TUNEL (green) labeling in the femur at E14.5 (D) or E18.5 (E). Region between dashed lines is used for quantification of EdU labeling index. Scale bar: 200 µm.

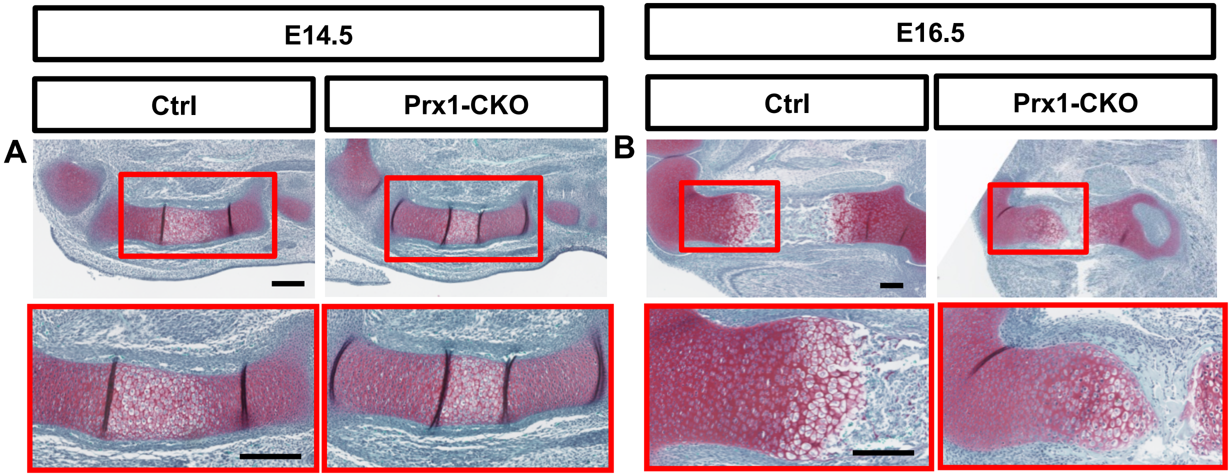

Supplementary Fig. 3. Glut1 is necessary for optimal production of proteoglycans. Representative images of Safranin O staining at E14.5 (A) or E16.5 (B). Boxed areas are shown at a higher magnification below. Scale bar: 200 µm.

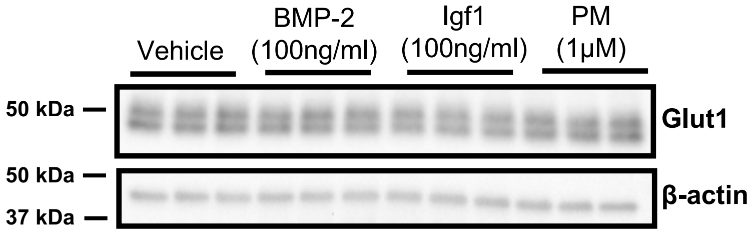

Supplementary Fig. 4. Lack of change in Glut1 levels after 6 hrs of treatment. Western blots of Glut1 in primary chondrocytes with indicated treatment for 6 hrs. β-actin used as loading control.

Western Blots for Fig 8A.

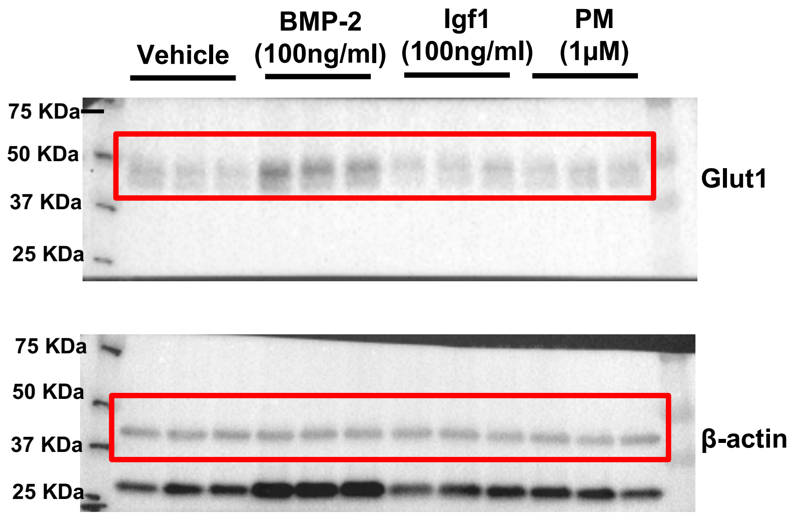

Supplementary Fig. 5. Uncropped Western blots for the study

Western Blots for Fig 8D.

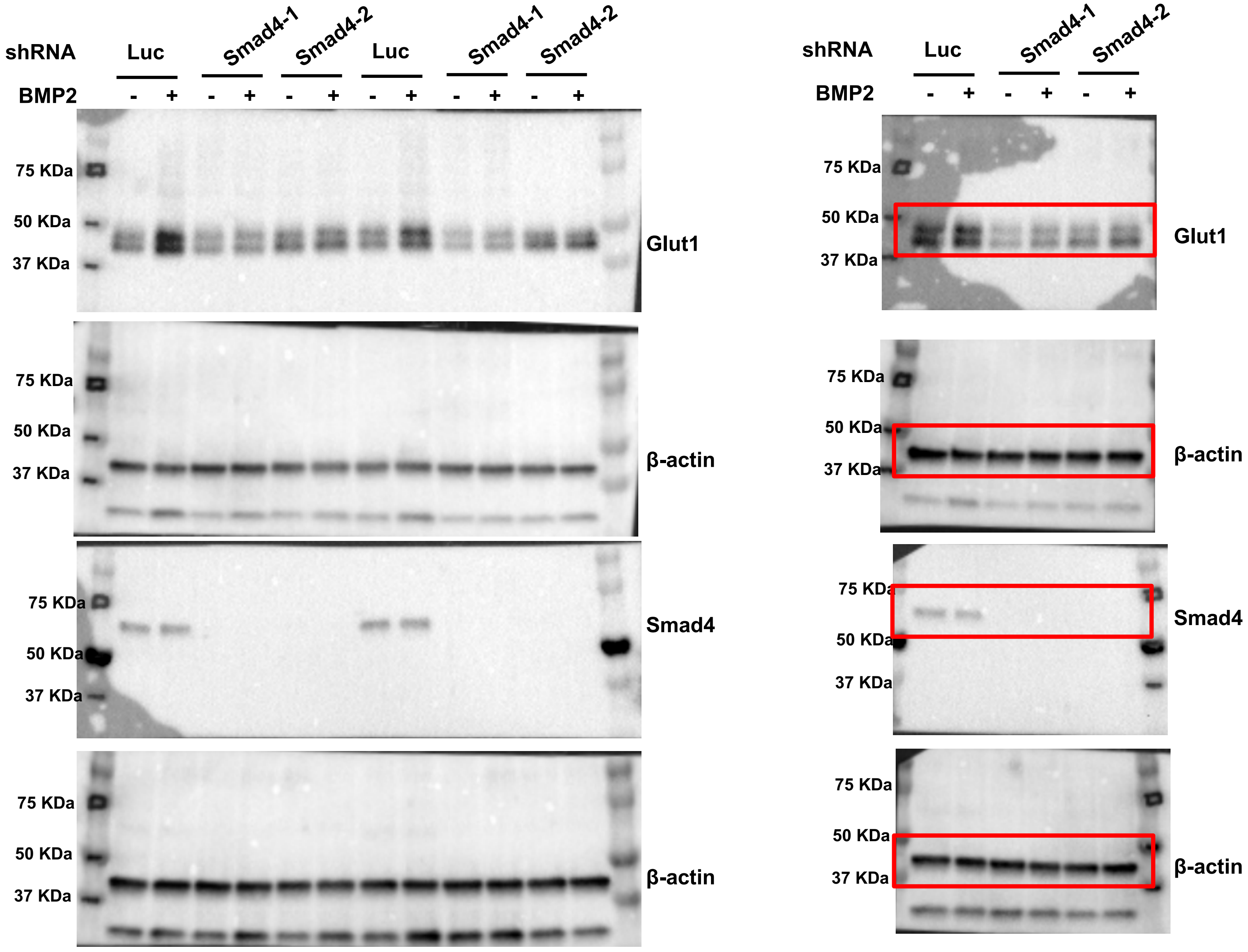

Supplementary Fig. 5. Uncropped Western blots for the study

Western Blots for Fig 8F

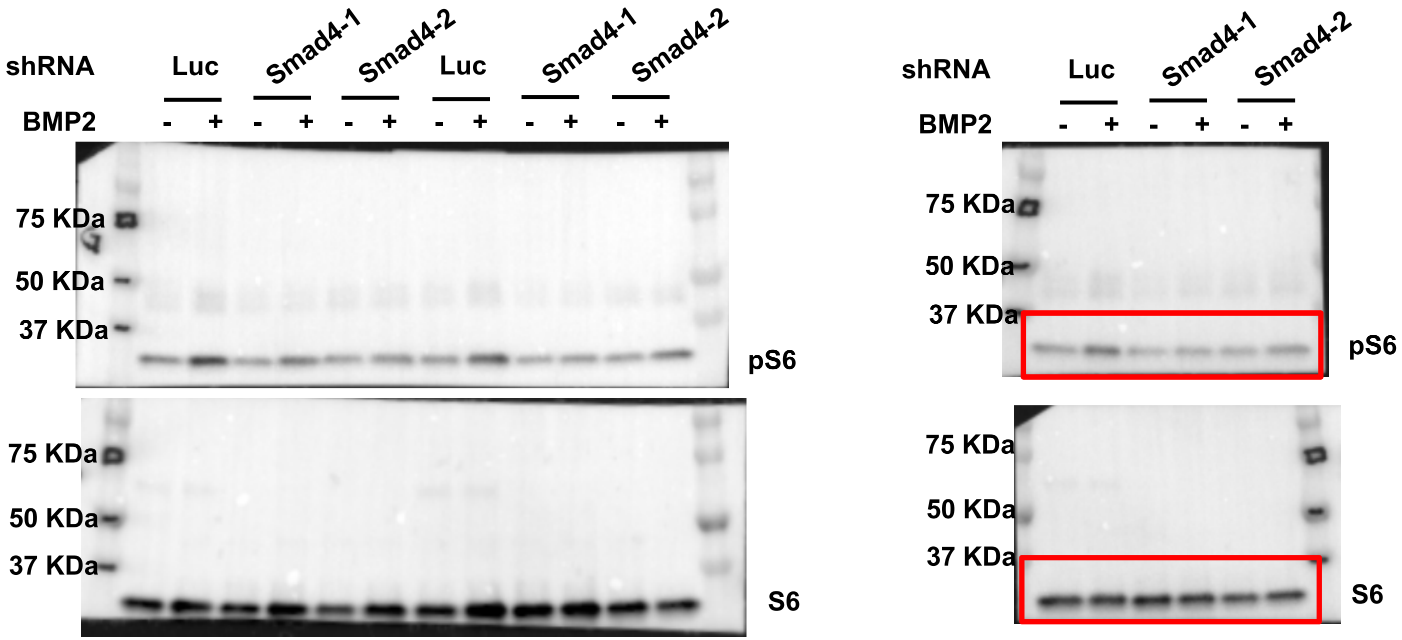

Supplementary Fig. 5. Uncropped Western blots for the study

Western Blots for Fig 9A

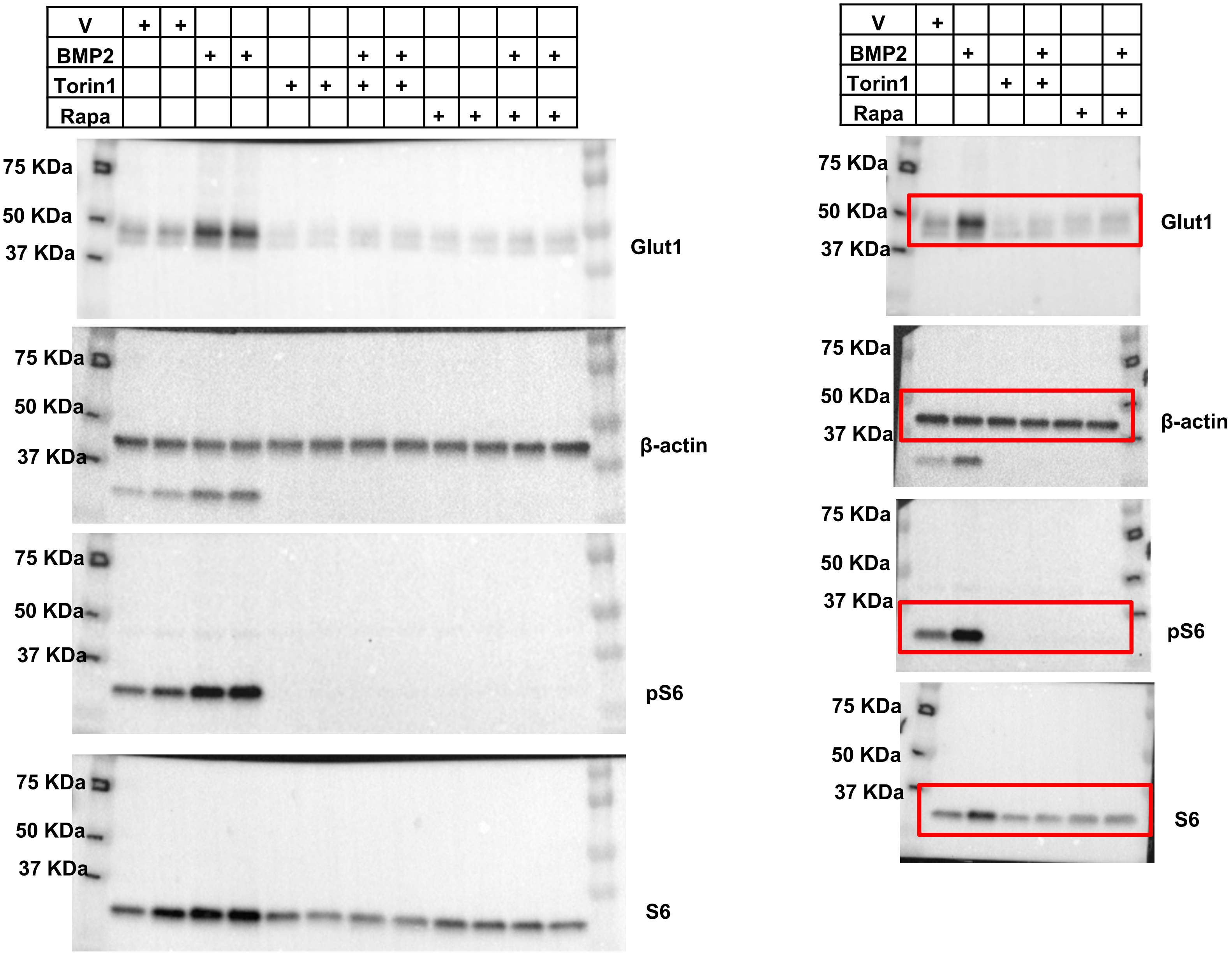

Supplementary Fig. 5. Uncropped Western blots for the study

Western Blots for Fig 9C

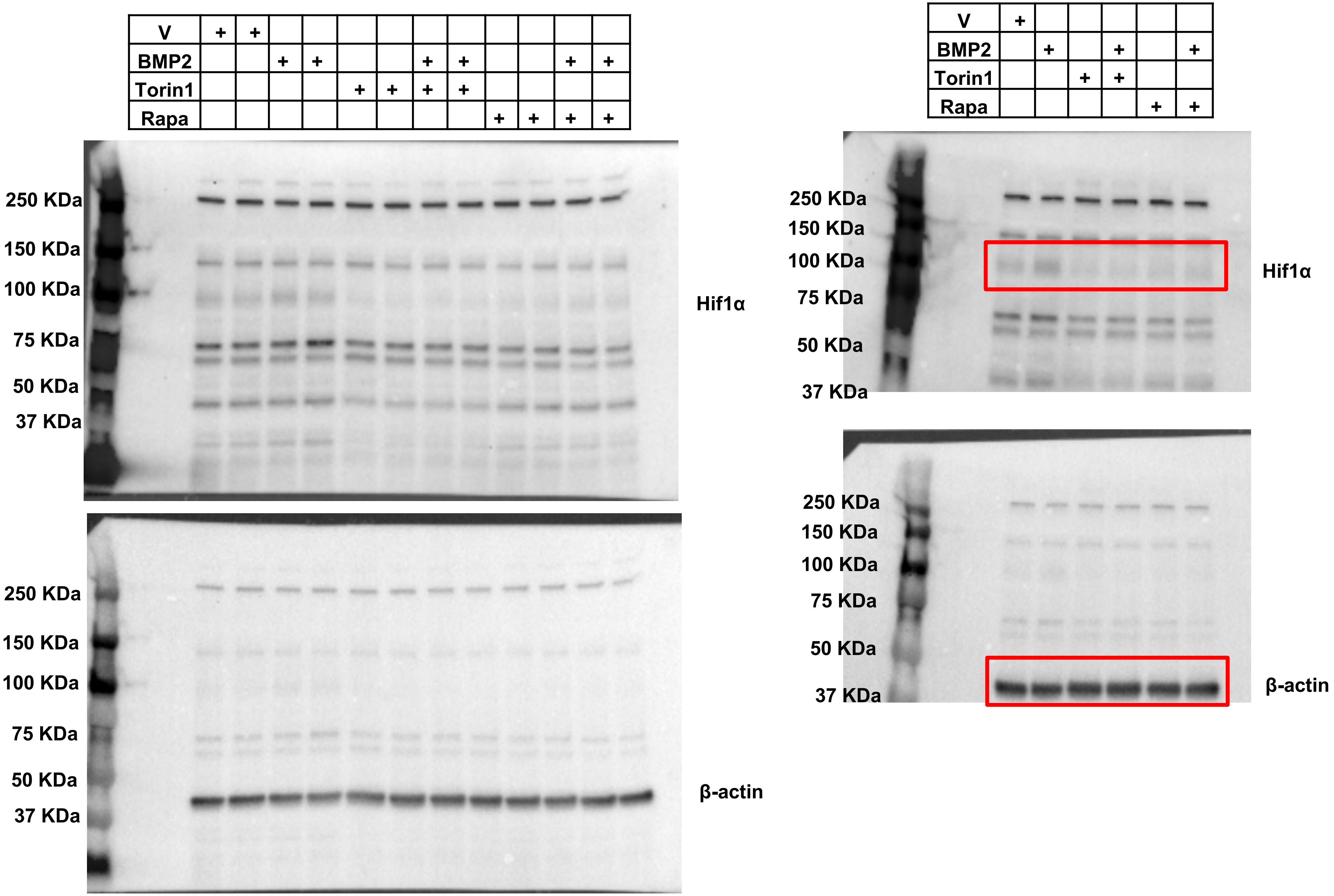

Supplementary Fig. 5. Uncropped Western blots for the study

Western Blots for Fig 9D

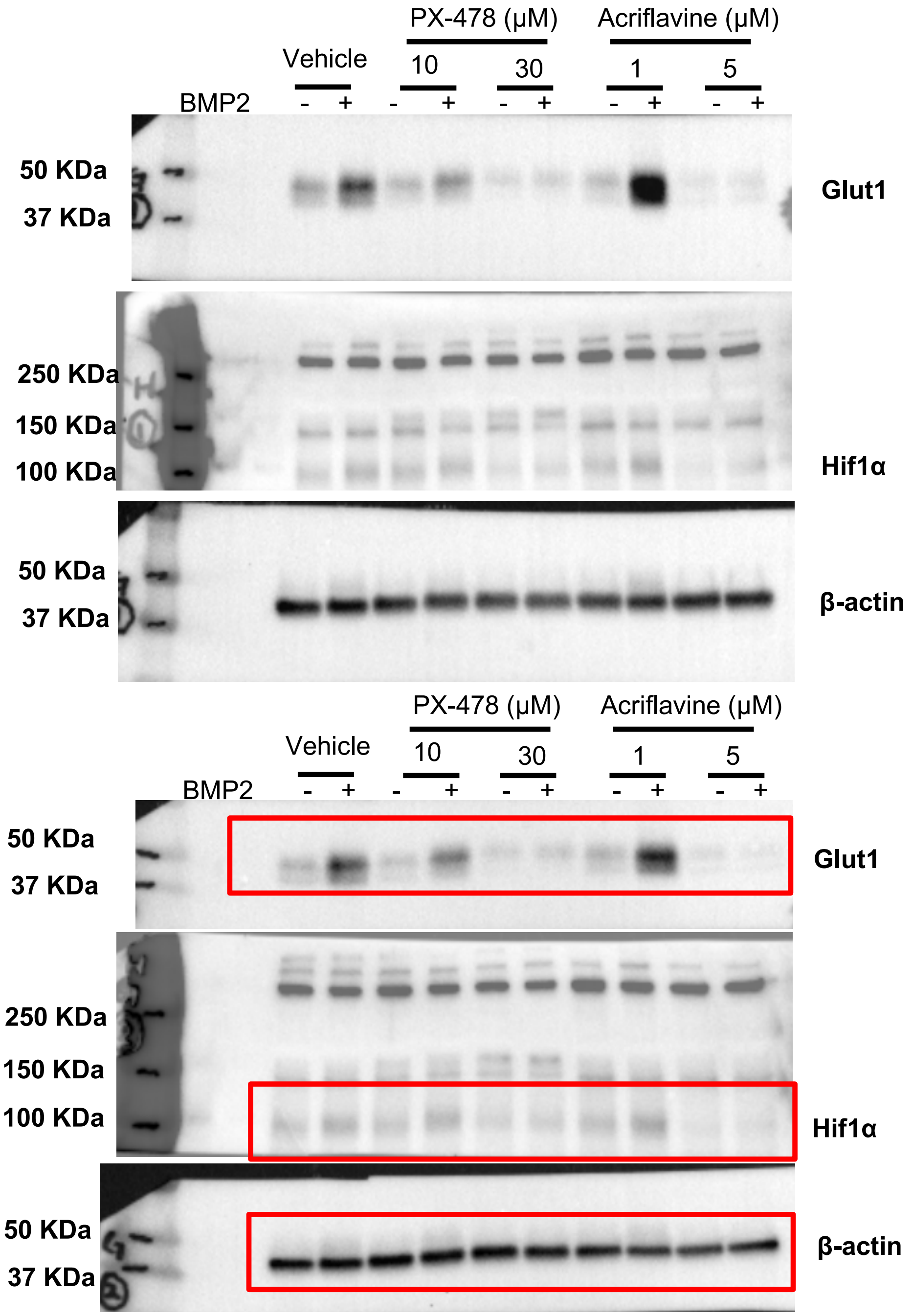

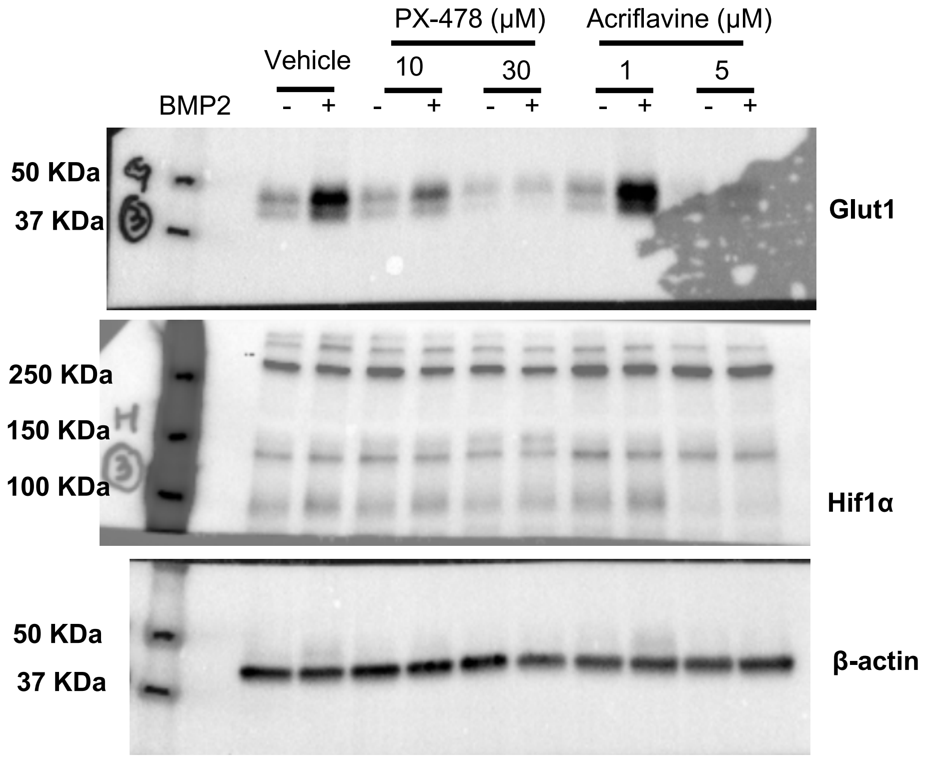

Supplementary Fig. 5. Uncropped Western blots for the study

Western Blots for Supplementary Fig. 4

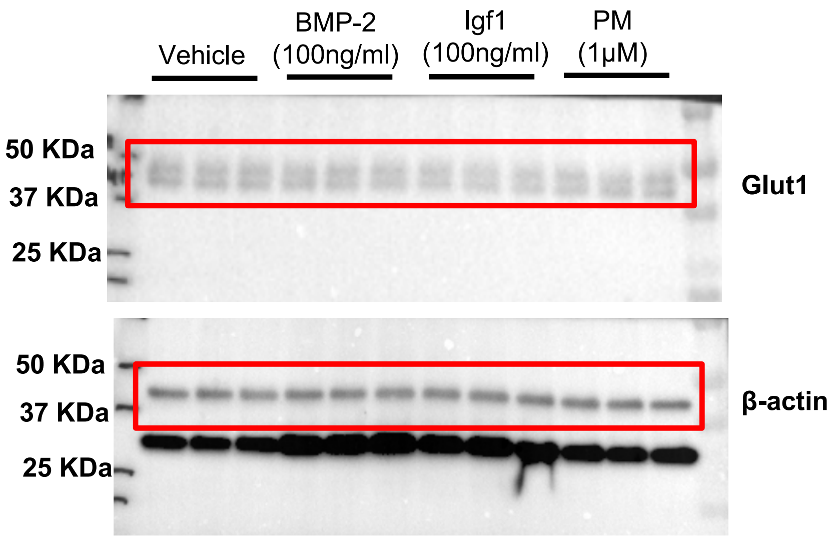

Supplementary Fig. 5. Uncropped Western blots for the study
